# Long non‐coding RNA MALAT1 targeting STING transcription promotes bronchopulmonary dysplasia through regulation of CREB

**DOI:** 10.1111/jcmm.15661

**Published:** 2020-08-18

**Authors:** Jia‐He Chen, Dan‐Dan Feng, Yu‐Fei Chen, Cai‐Xia Yang, Chen‐Xia Juan, Qian Cao, Xi Chen, Shuang Liu, Guo‐Ping Zhou

**Affiliations:** ^1^ Department of Pediatrics The First Affiliated Hospital of Nanjing Medical University Nanjing China; ^2^ Child Mental Health Research Center Nanjing Brain Hospital Affiliated to Nanjing Medical University Nanjing China

**Keywords:** cAMP response element–binding protein (CREB), Bronchopulmonary dysplasia (BPD), Metastasis‐associated lung adenocarcinoma transcript 1 (MALAT1), Stimulator of interferon genes (STING)

## Abstract

Bronchopulmonary dysplasia (BPD) is a severe complication of preterm infants characterized by increased alveolarization and inflammation. Premature exposure to hyperoxia is believed to be a key contributor to the pathogenesis of BPD. No effective preventive or therapeutic agents have been created. Stimulator of interferon gene (STING) is associated with inflammation and apoptosis in various lung diseases. Long non‐coding RNA MALAT1 has been reported to be involved in BPD. However, how MALAT1 regulates STING expression remains unknown. In this study, we assessed that STING and MALAT1 were up‐regulated in the lung tissue from BPD neonates, hyperoxia‐based rat models and lung epithelial cell lines. Then, using the flow cytometry and cell proliferation assay, we found that down‐regulating of STING or MALAT1 inhibited the apoptosis and promoted the proliferation of hyperoxia‐treated cells. Subsequently, qRT‐PCR, Western blotting and dual‐luciferase reporter assays showed that suppressing MALAT1 decreased the expression and promoter activity of STING. Moreover, transcription factor CREB showed its regulatory role in the transcription of STING via a chromatin immunoprecipitation. In conclusion, MALAT1 interacts with CREB to regulate STING transcription in BPD neonates. STING, CREB and MALAT1 may be promising therapeutic targets in the prevention and treatment of BPD.

## 1 INTRODUCTION

Bronchopulmonary dysplasia (BPD) is a serious chronic complication and a leading cause of respiratory morbidity. It requires supplemental oxygen or assisted ventilation, occurring almost exclusively in preterm infants.^1^ Poor outcomes even persist into their adulthood, like cardiovascular and chronic respiratory damage, neurocognitive disorder and growth failure.^2^, ^3^, ^4^ However, the pathogenesis of BPD is not fully understood. Currently, few evidence‐based strategies on BPD prevention and treatment are available.

Stimulator of interferon genes (STING; encoded by *TMEM173*; also known as MPYS and MITA) is a transmembrane protein mainly located on the endoplasmic reticulum of endothelial cells, epithelial cells as well as macrophages and dendritic cells (DCs).^5^, ^6^ STING is involved in lung inflammation and cell apoptosis, two processes indispensable in preterm BPD pathogenesis.^7^, ^8^, ^9^, ^10^ However, no research has gone further into the relevancy between STING and BPD.

Interestingly, our previous study has found that CREB is related to STING in other diseases. Cyclic adenosine monophosphate (cAMP) response element–binding protein (CREB) is activated ensuing phosphorylation in response to cAMP.^11^ CREB can function as a transcription factor to regulate cellular gene expression through phosphorylation at serine residue 133.^12^ Many studies have found that CREB is associated with various inflammation‐related signalling pathways, such as AMPK, Akt and STAT3 pathways.^13^, ^14^, ^15^ Hence, we speculate that CREB might participate in the development of BPD. Surprisingly, investigators verified that long non‐coding RNA MALAT1 could regulate retinal neurodegeneration through CREB signalling.^16^ Long non‐coding RNAs (lncRNAs) is a family of non‐coding RNAs with a sequence of exceeding 200 nucleotides.^17^ In this family, metastasis‐associated lung adenocarcinoma transcript 1 (MALAT1; also called NEAT2) shows a high expression in metastatic lung cancer.^18^ It is now well established that MALAT1 is involved in several physiopathological process, including inflammation.^19^ Preceding research showed that MALAT1 could promote cell apoptosis and inhibit cell proliferation in various diseases.^20^, ^21^ Interestingly, much literature has validated that MALAT1 expression was elevated in BPD patients, implying its participation in the pathogenesis in BPD.^22^ Nevertheless, the underlying mechanism remains largely obscure.

Herein, this study was designed to explore the roles of STING, CREB and MALAT1 in the pathogenesis and development of BPD, hoping to provide a new therapeutic target in the treatment of BPD infants.

## 2 MATERIALS AND METHODS

### 2.1 Subjects and sample collection

A total of 57 newborns with bronchopulmonary dysplasia (BPD) cases and unrelated healthy controls from the First Affiliated Hospital of Nanjing Medical University were enrolled on our study. The diagnosis of BPD was according to the paediatrician's diagnosis based on the National Institute of Child Health and Human Development (NICHD, 2000) guidelines.^23^, ^24^ Both 30 BPD infants’ and 27 normal controls’ blood were collected at 36 weeks post‐menstrual age (PMA). BPD severity was classified based on oxygen use at 36 weeks PMA, with a scale of mild (room air), moderate (<30% supplemental oxygen) and severe (>30% supplemental oxygen or positive pressure) according to National Heart, Lung, and Blood Institute (NHLBI) workshop definition.^24^ Infants with proven sepsis evidenced by a positive blood culture, major congenital anomalies or perinatal asphyxia were excluded from the study. Peripheral blood mononuclear cell (PBMC) specimens were obtained from the cases and controls with appropriate parents’ informed consent before participation. This study was approved by the Institutional Research Ethics Committee of the First Affiliated Hospital of the Nanjing Medical University. We announced that the study was based on the Helsinki Declaration of the World Medical Association.

### 2.2 The hyperoxia‐based rat model of BPD

All procedures were approved by the Animal Care and Use Committee of Nanjing Medical University. All studies used newborn rats and litter sizes for each experiment were adjusted to 5 pups in every treatment group to minimize the impacts of differences in nutrition on lung development. Fifty premature rats were randomly divided into two groups: the hyperoxia group and normoxia group. Rat pups were maintained in paired chambers (21% or 85% oxygen) for 1‐, 3‐, 7‐, 14‐ or 21‐day exposure periods. Nursing dams were switched between normoxic and hyperoxic chambers every 24 hours to limit oxygen toxicity. In all experiments, rat pups were euthanized at selected time points with intraperitoneal pentobarbital, and lung tissues were processed for RNA, protein and histology evaluation.

### 2.3 Cell culture and reagents

Human embryonic kidney 293 cells (HEK293), Human type II alveolar lung epithelium cells (A549) and human bronchial epithelium cells (Beas‐2B) were obtained from the American Type Culture Collection (ATCC). Cells were cultured in Dulbecco's modified Eagle's medium (DMEM) containing 10% heat‐inactivated foetal bovine serum (FBS), penicillin (100 unit/mL) and streptomycin (100 mg/mL) at 37℃ supplied with 5% CO2.

### 2.4 Plasmids and small interfering RNA (siRNA)

Transcriptional start site (TSS) of human STING promoter was set as +1 according to our previous study and the STING genomic DNA fragment (pGL‐126/+1) was inserted into the pGL3‐Basic vector (Promega, USA) designated as pGL‐126/+1. Mutations of the CREB‐binding sites on the STING minimal promoter were constructed as previously described. The pcDNA3.1‐STING and pcDNA3.1‐CREB expression plasmid as well as pcDNA3.1‐basic vector are kept by our laboratory. The double‐stranded siRNAs were designed and synthesized by the GenePharma company (Shanghai, China). The sequences targeted in the CREB and STING mRNA, as well as the lncRNA MALAT1 and negative control sequences were listed as follows:

CREB: 5′‐AGUAAAGGUCCUUAAGUGCTT‐3′

STING: 5′‐CGAAAUAACUGCCGCCUCATT‐3′

MALAT1: 5′‐GAGCAAAGGAAGUGGCUUATT‐3′

Control: 5′‐UUCUCCGAACGUGUCACGU‐3′.

### 2.5 Cell transfection and dual‐luciferase reporter assays

Transient transfections were carried out into A549, Beas‐2B and HEK293 cells by using Lipofectamine™ 3000 (Invitrogen) according to the manufacturer's protocol. Cells were seeded into 96‐well plates (1.5 × 10^4^/well) 24 hours before transfection. Then, 100 ng of each luciferase containing plasmid together with 4 ng of a control pRL‐TK plasmid were cotransfected into cells. Luciferase assay was conducted 24 hours after transfection by a Dual Reporter Assay System (Promega, USA) and TD‐20/20 Turner Designs Luminometer according to the manufacturer's suggestion. All results were representative of at least three independent experiments performed in triplicate.

### 2.6 RNA extraction and quantitative real‐time polymerase chain reaction(qRT‐PCR)

Total RNA was extracted using RNAiso Plus (Takara, Japan) according to the manufacturer's protocol and was then reverse transcribed into first‐strand cDNA by PrimeScript RT reagent Kit with gDNA Eraser (Takara, Japan). Quantitative real‐time PCR (qRT‐PCR) was performed with SYBR Green I Master Mix (Takara, Japan) in the StepOne Plus Real‐Time PCR System (Thermo Fisher Scientific, USA). The specificity of amplification was confirmed by a melting curve. Each sample was analysed in triplicate and was normalized to the expression level of human β‐actin. The relative mRNA expression level was calculated with 2‐ΔΔCt method. The primer pairs used for qRT‐PCR are listed below:

CREB: 5′‐CATTAACCATGACCAATGCAG‐3′(sense),

5′‐CTGTGCGAATCTGGTATGTTT‐3′ (antisense);

STING: 5′‐GGGCTGGCATGGTCATATTA‐3′(sense),

5′‐TACTCAGGTTATCAGGCACC‐3′(antisense);

MALAT1: 5′‐AAGATGAGGGTGTTTACG‐3′(sense),

5′‐AAGCCTTCTGCCTTAGTT‐3′(antisense);

β‐actin: 5′‐AAAGACCTGTACGCCAACAC‐3′(sense),

5′‐GTCATACTCCTGCTTGCTGAT‐3′(antisense).

### 2.7 Western blotting analysis

To determine the levels of protein expression, protein of A549 and Beas‐2B cells was extracted by a Total Protein Extraction Kit (Keygentec, China). The concentrations were measured by a BCA Protein Assay Kit (Pierce, USA). Samples were run on 8% and 12% SDS‐PAGE gels, and then, separated proteins were transferred onto PVDF membranes (Millipore, USA). The membranes were blocked for 2 hours with 5% dry milk in Tris‐buffered saline plus Tween 20 (TBST, pH 7.4) and then immunodetected with rabbit anti‐STING (1:1000), rabbit anti‐CREB (1:500), rabbit anti‐p‐CREB (1:500), rabbit anti‐β‐actin (1:1000), rabbit anti‐PARP (1:1000) and rabbit anti‐caspase‐3 (1:1000) antibodies overnight at 4°C. STING antibodies was from ProteinTech (USA), CREB and p‐CREB antibodies were purchased from Santa Cruz (USA), β‐actin antibodies was got from Abcam (UK) and other antibodies were obtained from Cell Signaling Technology (USA). After three washes with TBST, membranes were treated with a horseradish peroxidase (HRP)‐labelled secondary antibody (1:10 000, Santa Cruz) for 1 hour at room temperature. Signals of membranes were visualized by enhanced chemiluminescence (ECL) detection systems (Pierce, USA) with a Bio‐Rad ChemiDoc XRS (USA).

### 2.8 Chromatin immunoprecipitation (ChIP)

The chromatin immunoprecipitation assay was performed by using the Magna ChIP G Chromatin Immunoprecipitation Kit (Millipore, USA) following the manufacturer's instructions. In brief, about 1 × 10^7^ A549 cells were fixed in 1% formaldehyde and the cell lysates were sonicated into DNA fragments in the range of 200‐1000 bp. The antibody used in the ChIP assays was an anti‐CREB antibody (Abcam, UK), and an anti‐IgG control antibody (Millipore, USA) was used as a negative control. The purified DNA from input or immunoprecipitated samples were assayed by qPCR with SYBR Green SYBR Green I Master Mix (Takara, Japan) after reverse cross‐linking and DNA purification. The primers used were listed as follows: 5′‐GCTCCTACCTAATATCATCCCC‐3′ (sense); 5′‐AGTTATTTCCGGTAACAAGAGC‐3′ (antisense).

### 2.9 Flow cytometry

To access apoptosis levels of different groups, we used a Annexin V Alexa Fluro 647‐A apoptosis detection kit (Fcmacs, China) following the manufacturer's instructions. In brief, different groups of A549 and Beas‐2B cells were seeded in a 6‐well plate, transfected with siRNAs or plasmids and then challenged with hyperoxia conditions. After 48 hours, cells were harvested and rinsed twice with PBS at 4°C. After 1 × binding buffer was used to resuspend cells, cells were incubated in the dark with Annexin V Alexa Fluro 647 and propidium iodide (PI) for approximately 15 min at room temperature. C6 Flow Cytometer™ system (BD Biosciences, CA, USA) was employed to analyse the apoptotic rate of cells.

### 2.10 Cell proliferation assay

Cell proliferation assays were carried out using Cell Counting Kit‐8 (CCK‐8) assay (Synthgene, China) according to the manufacturer's instructions. Briefly, cells were seeded in a 96‐well plate in 5 replicates at a density of 3000 cells with 100 μL of culture medium per well. Then, cells were put into a high oxygen incubator. At the indicated time point, 10 μL of the CCK‐8 reagent was added to each well, and cells were incubated for an additional 4 hours at 37°C. Viable cells were counted by recording the absorbance at a reference wavelength of 450 nm with a microplate reader (Varioskan Flash; Thermo Scientific, Waltham, MA, USA). The experiments were repeated in triplicate on separate occasions independently.

### 2.11 Haematoxylin and eosin staining (H&E)

H&E staining was performed on deparaffinized tissue sections according to standard protocols. Sections were cut into 5‐μm slices for pathological evaluation and observation under a microscope (Olympus, Tokyo, Japan). The lung tissues were stained with HE method in order to assess lung histological changes. The radial alveolar count (RAC) was determined using the method developed by Emery and Mithal^25^ in order to evaluate the development of pulmonary alveoli. The RAC of each section was evaluated by two independent pathologists who were blinded to the experimental design.

### 2.12 Immunohistochemistry (IHC)

Specimens were stained with antibodies for STING (1:100), CREB (1:100), α‐SMA (1:320) and E‐Cadherin (1:100). STING and E‐Cadherin antibodies were from ProteinTech (USA), CREB antibodies was purchased from Santa Cruz (USA) and other antibodies were obtained from Cell Signaling Technology (USA). The sections were heated at 70°C for 1 hour, deparaffinized in xylene twice and rehydrated through a gradient concentration of alcohol five times. To block endogenous peroxidase and reduce non‐specific reaction, sections were stained with 3% hydrogen peroxide and normal bovine serum, then incubated with primary antibody against STING or CREB overnight at 4°C. The slides were then incubated with horseradish peroxidase (HRP)‐conjugated secondary antibody at 37°C for 10 minutes. Signals were detected by diaminobenzidine (DAB) solution. Finally, sections were counterstained with haematoxylin, dehydrated and mounted. Images were acquired for examination. Scoring was comprehensively conducted depending on the staining intensity (0 for no staining, 1 for weak staining, 2 for moderate staining and 3 for strong staining) and percentage of positively stained cells (0 for 0%‐5% of cells, 1 for 6%‐25% of cells, 2 for 26%‐50% of cells, 3 for 51%‐75% of cells and 4 for 76%‐100% of cells). The product of both grades was calculated as the final expression score.

### 2.13 Immunofluorescence (IF)

Cells on glass slides were fixed by neutral paraformaldehyde (4%) for 30 minutes and permeabilized using PBS containing 0.01% Triton X‐100 for 15 minutes. Next, cells were incubated with rabbit anti‐STING (1:50) antibody or rabbit anti‐CREB (1:50) antibody at 37°C for 2 hours, followed by incubation with the appropriate secondary antibody (Jackson, USA) for 1 hour at 37℃. STING antibody was from ProteinTech (USA) and CREB antibody was purchased from Santa Cruz (USA). Additionally, cells were incubated with 4′,6‐diamidino‐2‐phenylindole (DAPI) as a nuclear counterstain. A confocal laser scanning microscope (Olympus BX43, Japan) was used for confocal microscopy.

### 2.14 Statistical analysis

The results were presented as the mean ± standard deviation (SD), and experiments were repeated at least three independent experiments. Statistical analysis was performed using GraphPad Prism 7 and SPSS 22.0. Results were considered statistically significant at *P < 0.05, **P < 0.01, ***P < 0.001.

## 3 RESULTS

### 3.1 STING and MALAT1 were highly expressed in PBMC specimens of BPD patients

**FIGURE 1:**
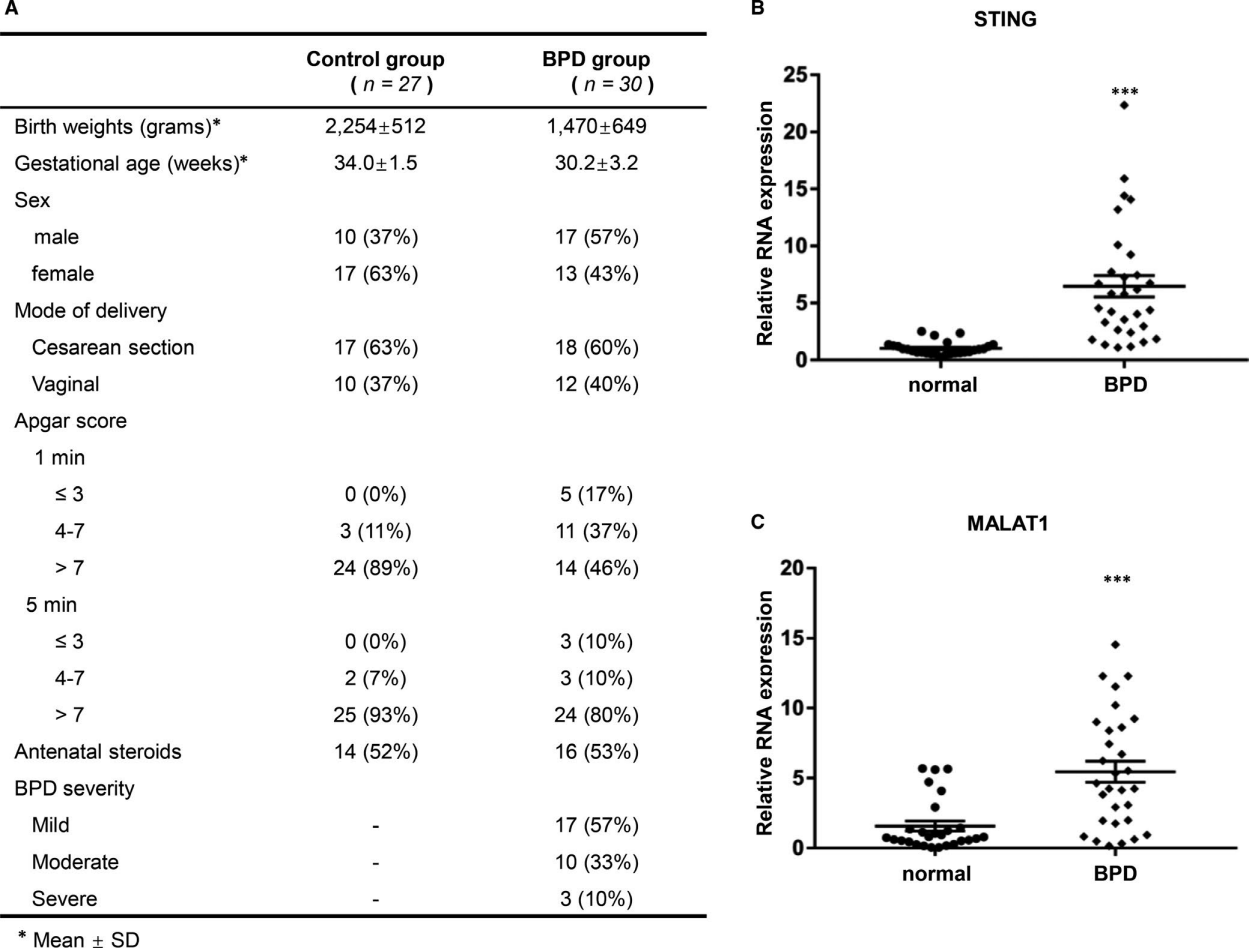
STING and MALAT1 were highly expressed in PBMC specimens of BPD infants compared with controls. A, Characteristics of enrolled infants. B and C, mRNA levels of STING and MALAT1 between controls and BPD infants were analysed by qRT‐PCR. Data were determined using Student's t test and expressed as mean ± SD of three independent experiments (***P < 0.001)

The basic characteristics of subjects and controls are listed in Figure 1A. STING and MALAT1 expression levels were analysed in PBMC specimens. The mRNA levels of both STING and MALAT1 were greatly increased in BPD patients, compared with those in normal subjects (Figure 1B: mean ± SD =1.022 ± 0.1106 vs 6.459 ± 0.9367, *P* < 0.001; Figure 1C: mean ± SD = 1.578 ± 0.3565 vs 5.451 ± 0.75, *P* < 0.001).

### 3.2 STING and MALAT1 were highly expressed in the lung tissue of BPD rats

**FIGURE 2:**
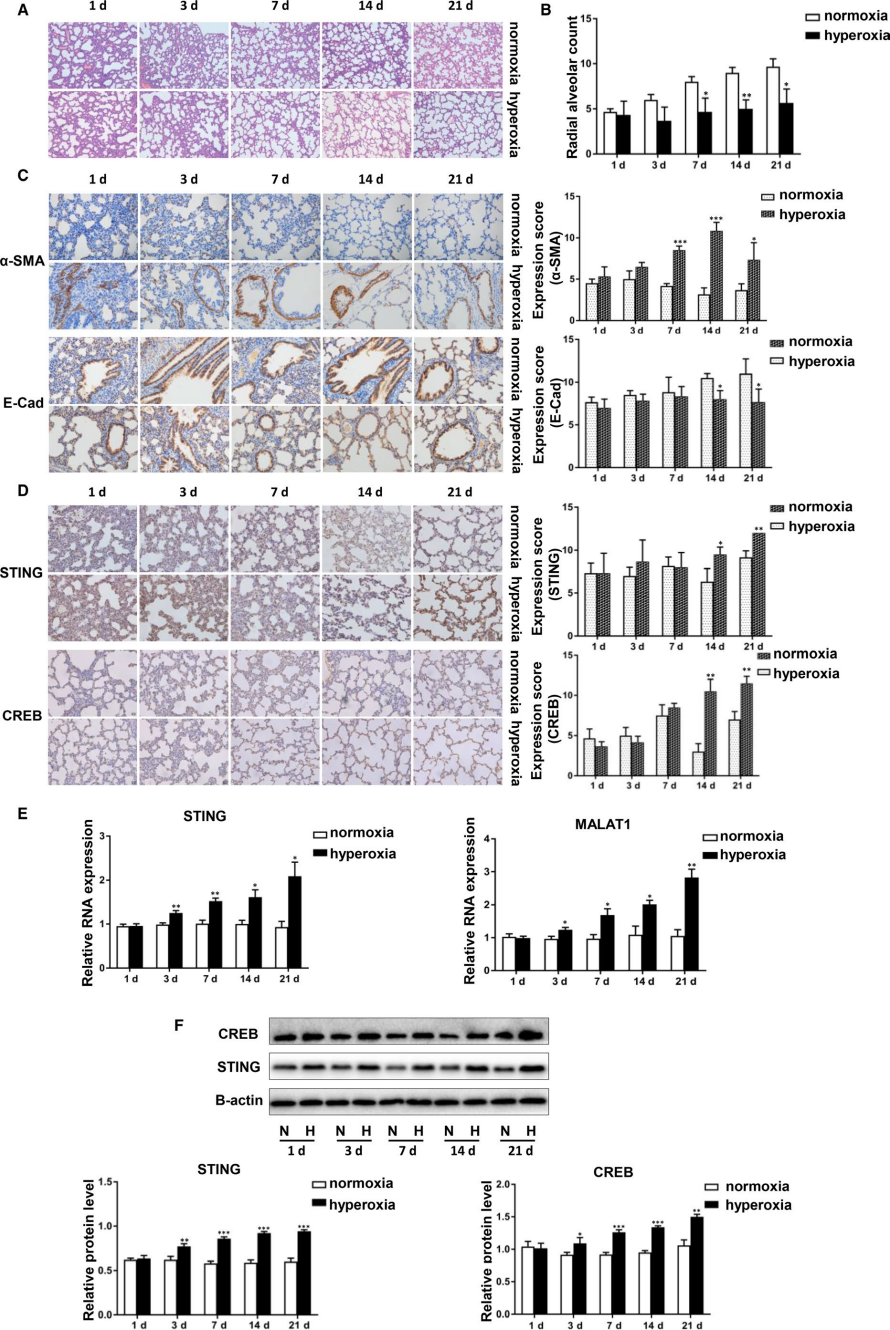
STING and MALAT1 were highly expressed in the lung tissue of BPD rats. A, H&E‐stained lung sections in hyperoxia‐exposed lungs and normoxia lungs from Day 1 to 21 rats. B, Comparison of radial alveolar count (RAC) between the two groups. C, The expression of α‐SMA and E‐Cadherin in hyperoxia tissues and normoxia tissues detected by IHC. The data have been quantified by expression score. D, The expression of STING and CREB in hyperoxia tissues and normoxia tissues detected by IHC. The data have been quantified by expression score. E, The mRNA levels of STING and MALAT1 in hyperoxia tissues and normoxia tissues were analysed by qRT‐PCR. F, STING and CREB protein levels were determined by Western blotting analysis and protein grey were scanned in hyperoxia and normoxia rats. All measurements are shown as the mean ± SD from three independent experiments (*P < 0.05, **P < 0.01, ***P < 0.001)

Rats exposed to hyperoxia had enlarged air spaces and fewer alveolar septa compared with those exposed to normoxia. Alveolar development in the hyperoxia group rats was inhibited (Figure 2A). RACs of the hyperoxia group were significantly lower than those in the normoxia group. The RACs in the normoxia group gradually increased over time after birth, becoming higher on 7, 14 and 21 days as compared to 1 day (Figure 2B). The E‐Cadherin and α‐SMA in the lung tissues of rats, which were markers of BPD, have been assessed by immunohistochemical staining. α‐SMA was up‐regulated whereas E‐Cadherin was down‐regulated in hyperoxia‐exposed rats (Figure 2C). Then, the expression of CREB and STING in the lung tissues of rats were assessed. A gradual upward trend was found in STING and CREB protein expression in hyperoxia‐exposed rats (Figure 2D). We also examined STING protein levels by Western blotting and mRNA levels by qRT‐PCR in lung tissues. Compared with that in the normoxia group, the mRNA and protein levels of STING expression in the hyperoxia group were gradually increased from Day 1 to 21 after birth. Meanwhile, the mRNA level of MALAT1 was also increased in premature rats exposed to hyperoxia, which is similar to that of STING (Figure 2E,F).

### 3.3 STING and MALAT1 were up‐regulated in hyperoxia‐stimulated lung epithelial cells

**FIGURE 3:**
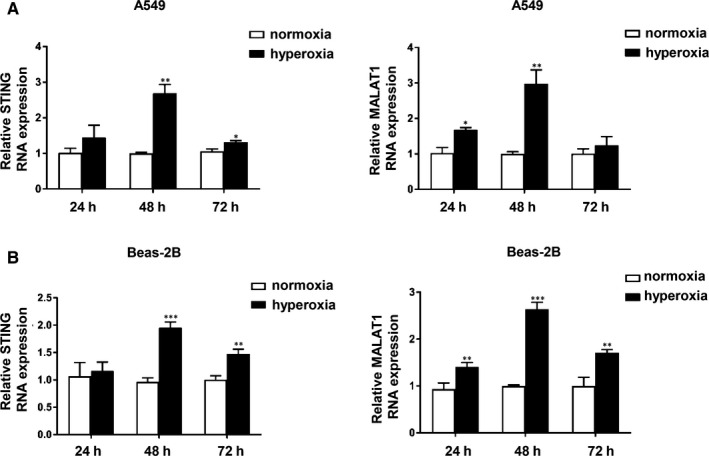
STING and MALAT1 were up‐regulated in hyperoxia‐stimulated lung epithelial cells. A and B, A549 and Beas‐2B cells were treated with hyperoxia or normoxia for 24 h, 48 h and 72 h, respectively. qRT‐PCR was used to detect the mRNA levels of both MALAT1 and STING (*P < 0.05, **P < 0.01, ***P < 0.001)

Hyperoxia conditions is generally considered to be related to high incidence of BPD.^26^ Thus, we investigated the potential relevance of MALAT1 and STING in hyperoxia‐induced lung epithelial cells. A549 and Beas‐2B cells were treated with hyperoxia or normoxia for 24 hours, 48 hours and 72 hours, respectively. The mRNA levels of both MALAT1 and STING were significantly increased after 48 hours of hyperoxic stimulation and then decreased over time, indicating that MALAT1 might act with STING reciprocally (Figure 3A,B).

### 3.4 Silencing both STING and MALAT1 promoted the proliferation and repressed the apoptosis of cells

**FIGURE 4:**
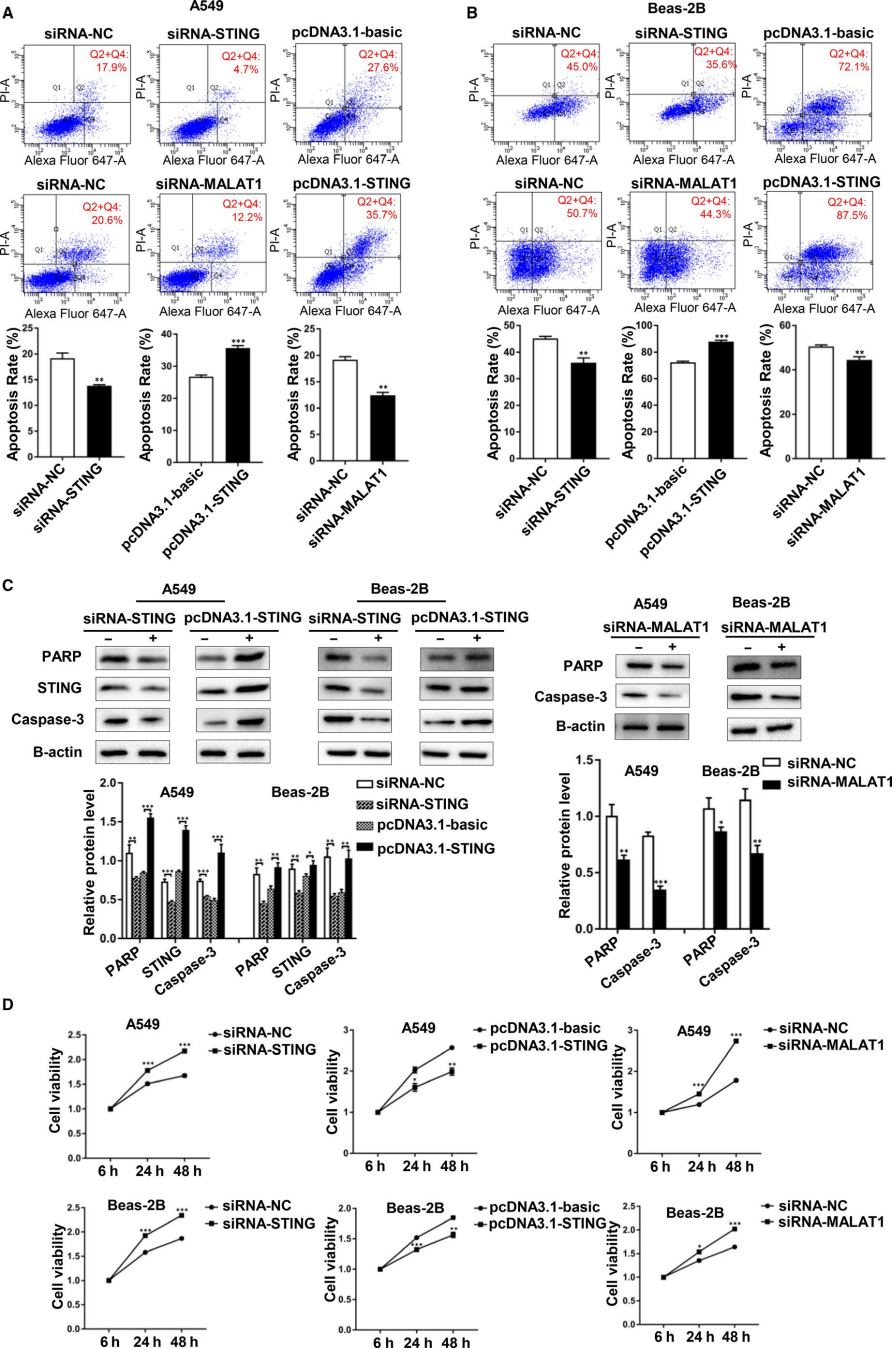
Silencing both STING and MALAT1 promoted the proliferation and repressed the apoptosis of cells. A, pcDNA3.1‐STING or the empty vector, siRNA‐STING or siRNA‐NC and siRNA‐MALAT1 or siRNA‐NC were transfected into A549 cells. The cell apoptosis rates were tested by a flow cytometry. Red numbers showed the apoptosis district and proportion. B, pcDNA3.1‐STING or the empty vector, siRNA‐STING or siRNA‐NC and siRNA‐MALAT1 or siRNA‐NC were transfected into Beas‐2B cells. C, Meanwhile, STING, PARP and caspase‐3 protein expression were detected by western blotting analysis. Protein levels have been quantified. D, Viability of A549 and Beas‐2B cells after silencing STING or MALAT1 or overexpressing STING was assessed by CCK‐8 assay at indicated times (*P < 0.05, **P < 0.01, ***P < 0.001)

We transfected STING siRNA, STING overexpression plasmids and MALAT1 siRNA into hyperoxia‐induced A549 and Beas‐2B cells. According to the results of flow cytometry, the apoptotic rate of cells decreased after STING or MALAT1 was knocked down, and then increased after STING was overexpressed (Figure 4A,B). According to the results of Western blotting, PARP and Caspase‐3 (two indicators of apoptosis) were down‐regulated after STING or MALAT1 was silenced, but up‐regulated after STING was overexpressed (Figure 4C). The results of CCK‐8 assay validated these effects of STING and MALAT1. We also discovered the increased viability in siRNA‐STING‐ or siRNA‐MALAT1‐treated cells, and decreased viability in pcDNA3.1‐STING‐treated cells (Figure 4D). In summary, silencing both STING and MALAT1 could promote the proliferation and repress the apoptosis of A549 and Beas‐2B cells.

### 3.5 Knockdown of MALAT1 suppressed expression and transcriptional promoter activity of STING in hyperoxia‐induced lung epithelial cells

**FIGURE 5:**
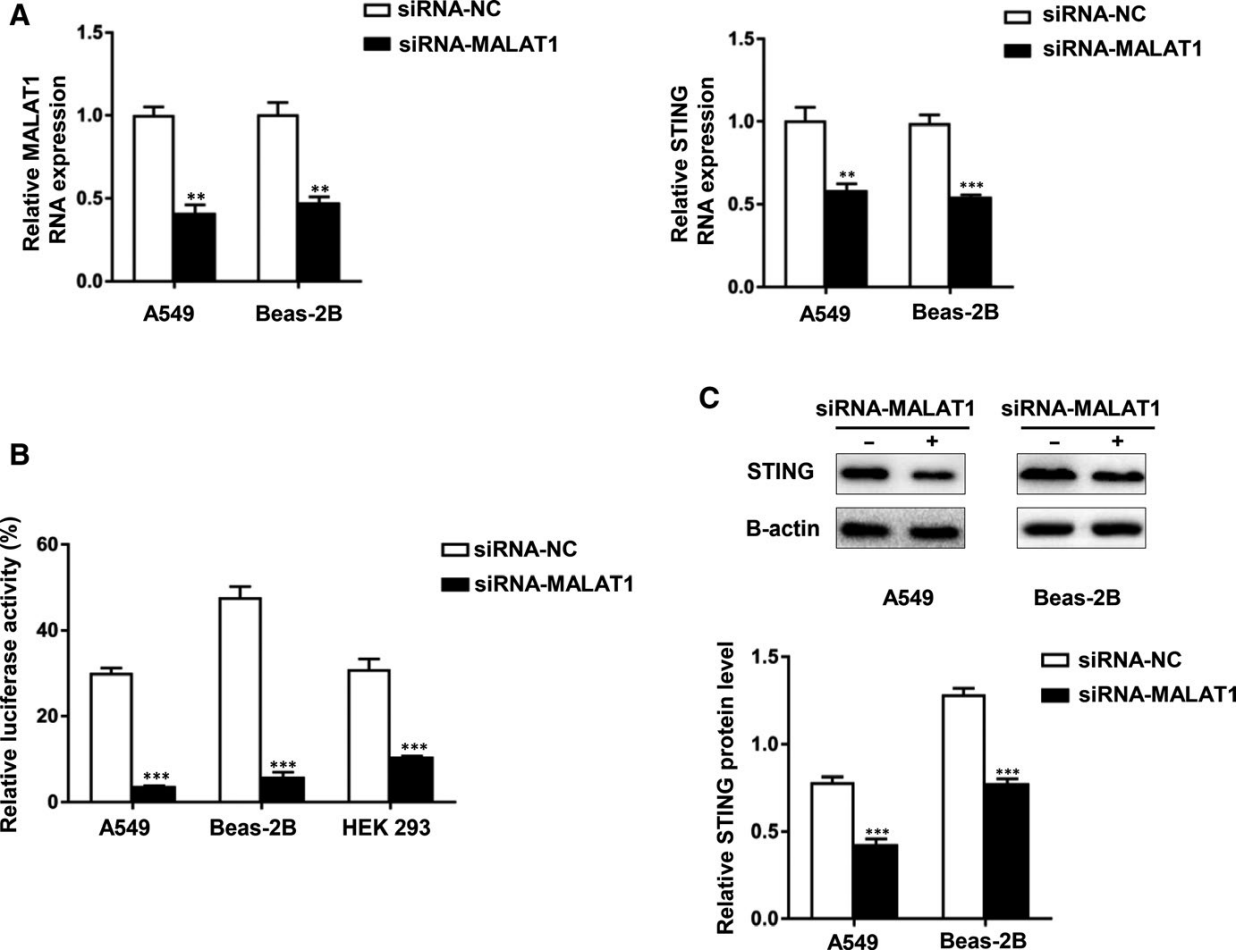
Knockdown of MALAT1 suppressed expression and transcriptional promoter activity of STING in hyperoxia‐induced lung epithelial cells. A, A549 and Beas‐2B cells were transfected by siRNA‐MALAT1 or siRNA‐NC. Relative RNA levels of MALAT1 and STING were analysed by qRT‐PCR. B, A549, Beas‐2B and HEK293 cells were cotransfected with the luciferase reporter plasmid containing the wild‐type STING promoter (pGL‐126/+1) and MALAT1 siRNA. Cells were harvested to measure luciferase activity (**P < 0.01, ***P < 0.001). C, A549 and Beas‐2B cells were transfected with MALAT1 siRNA or NC siRNA. STING protein level was detected by Western blotting analysis after stimulating in hyperoxia. Data are representative of three independent experiments

To investigate the role of MALAT1 in the regulation of STING expression, we knocked down MALAT1 into Beas‐2B and A549 cells and then exposed the cells to hyperoxia for 48 hours. The level of MALAT1 expression decreased in both cell lines. We observed that the mRNA level of STING was down‐regulated after the transfection with siMALAT1 by qRT‐PCR (Figure 5A). We also cotransfected plasmids pGL‐126/+1 and pRL‐TK together with MALAT1 siRNA into Beas‐2B, A549 and HEK293 cells to evaluate whether MALAT1 affects the promoter activity of STING. As expected, suppressing MALAT1 induced a decrease in luciferase activity of STING in the three cell lines (Figure 5B). Additionally, the STING protein level decreased in siRNA‐MALAT1‐treated cells compared with that in siRNA‐NC‐treated cells (Figure 5C). These data demonstrate that knocking down MALAT1 suppressed the expression and transcriptional promoter activity of STING in hyperoxia‐induced lung epithelial cells.

### 3.6 Hyperoxia‐induced STING transcription depended on CREB

**FIGURE 6:**
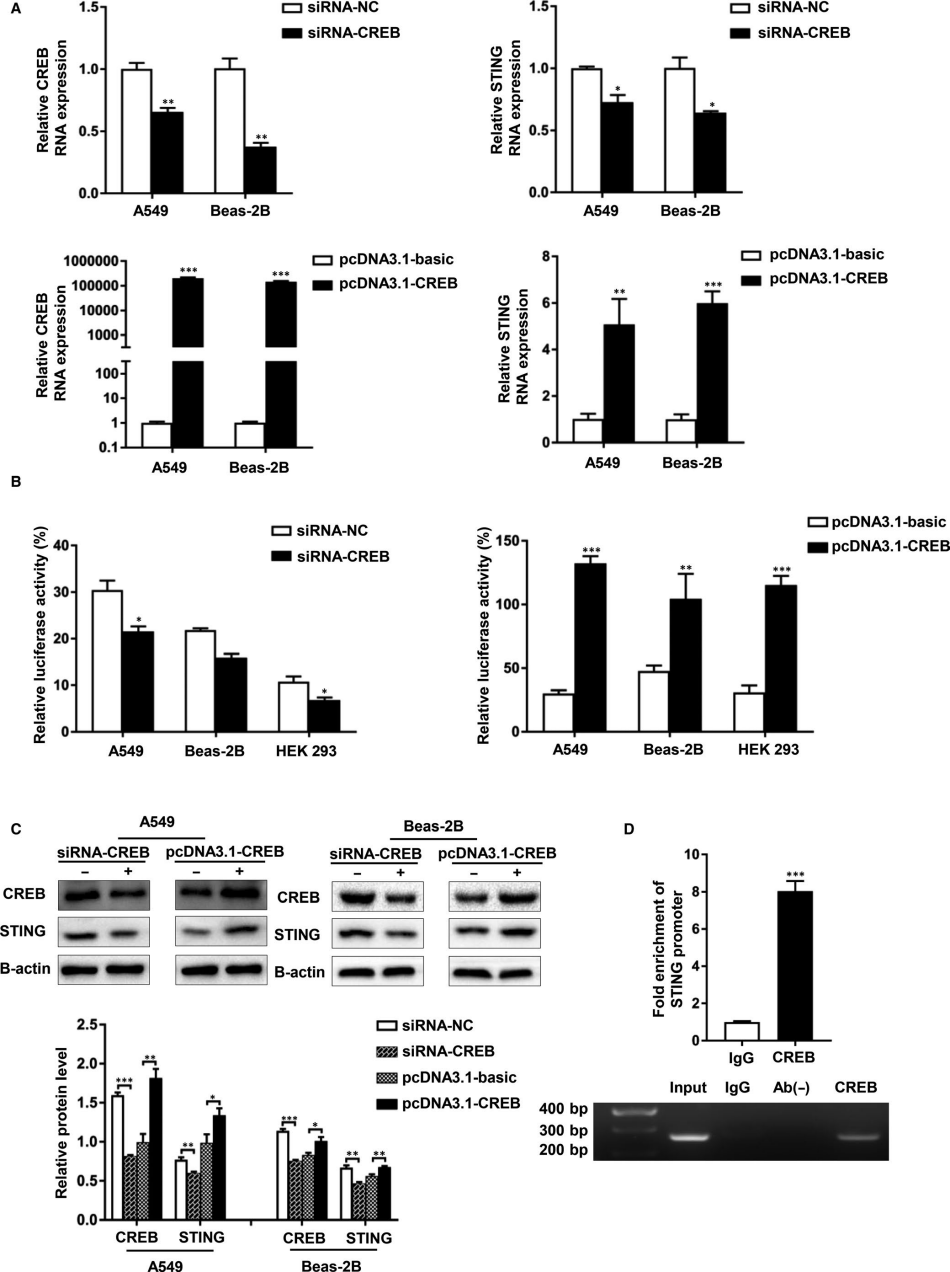
Hyperoxia‐induced STING transcription depended on CREB. A, A549 and Beas‐2B cells were transfected with CREB siRNA or pcDNA3.1‐CREB plasmids. CREB and STING RNA levels were analysed then. B, A549, Beas‐2B and HEK293 cells were transiently transfected with CREB expression plasmid or CREB siRNA and pGL‐126/+1. A dual‐luciferase assay was performed to detect the activity of STING promoter. C, A549 and Beas‐2B cells were transfected with siRNA‐CRAB or CREB overexpression plasmids. CREB and STING protein levels were monitored then. D, A representative gel image of PCR products obtained by ChIP. A549 cells were induced in hyperoxia. Anti‐CREB antibodies were used to precipitate proteins bound to the amplified sequence of the endogenous STING promoter. In contrast, no signal was apparent in the negative control. The chromatin fragments were PCR‐amplified using primers specific for the proximal CREB‐binding site of the STING promoter and presented on an agarose gel. The chromatin fragments were quantified by qRT‐PCR. Each experiment was performed in triplicate, and significant differences were determined with an unpaired t test (*P < 0.05, **P < 0.01, ***P < 0.001)

Through the cell immunofluorescence, we observed that STING was located mainly in the cytoplasm while CREB was located in both cytoplasm and nucleus (see Appendix 1). First, we transfected siRNAs targeting CREB (siRNA‐CREB) and overexpression plasmids (pcDNA3.1‐CREB) into the cell lines and exposed them to hyperoxia for 48 hours. We found that both the mRNA and protein levels of STING were down‐regulated after CREB knockdown and then up‐regulated after CREB overexpression (Figure 6A,C). Furthermore, we constructed a plasmid with the core region containing the sequence between −126 and +1 upstream of human STING promoter. To determine whether CREB directly regulates STING transcription, we cotransfected pcDNA3.1‐CREB plasmid or siRNA‐CREB with pGL‐126/+1 plasmid into A549, Beas‐2B and HEK293 cells under hyperoxia. As shown in figures, the overexpression of CREB increased the luciferase activity and siRNA‐CREB decreased this activity (Figure 6B). To confirm the binding of CREB to the STING promoter, we performed a ChIP assay for hyperoxia‐treated A549 cell extracts using anti‐CREB and anti‐IgG antibody. As is observed in figures, compared with the non‐specific IgG control antibody, the STING promoters and the CREB antibodies were significantly enriched (Figure 6D). Therefore, it was elucidated that CREB could directly bind to the promoter of STING to regulate its transcription. Based on these data, we concluded that hyperoxia‐induced STING transcription depended on CREB.

### 3.7 MALAT1 worked with CREB to modulate the transcription of STING

**FIGURE 7:**
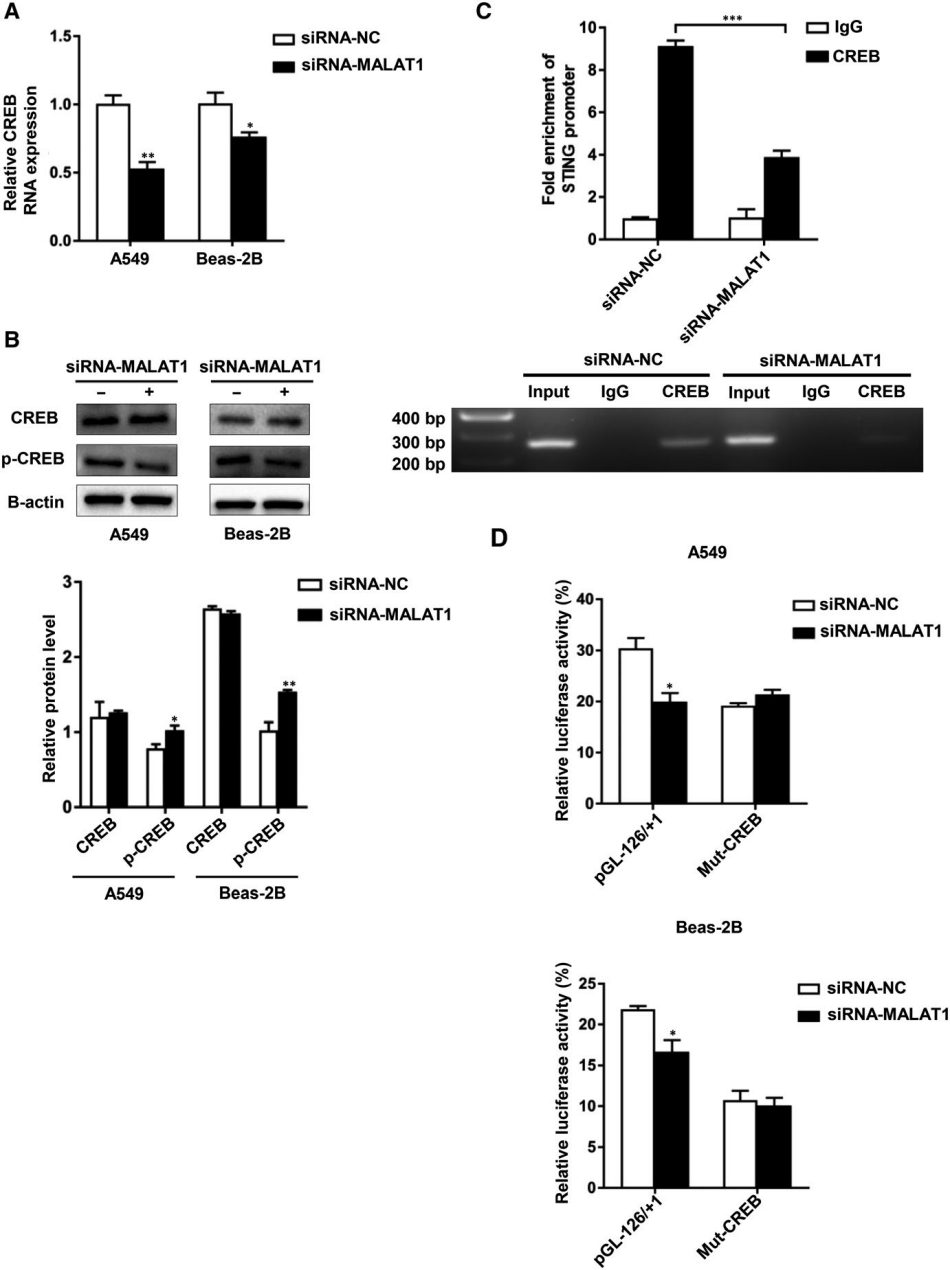
MALAT1 worked with CREB to modulate the transcription of STING. A, CREB relative RNA expression was analysed following the MALAT1 silencing. B, The protein level of CREB or p‐CREB was examined by Western blotting. Protein levels were quantified. C, A549 cells were treated with siRNA‐MALAT1 or siRNA‐NC in hyperoxia. Then a ChIP assay was performed as above. D, A549 and Beas‐2B cells were cotransfected with siRNA‐MALAT1 and pGL‐126/+1 or plasmids containing mutated CREB‐binding sites. Subsequently, the STING promoter activity was detected by a dual‐luciferase assay. Results were presented as the means ± SD of triplicates (*P < 0.05, **P < 0.01, ***P < 0.001)

We examined whether STING transcription is modulated via the MALAT1‐CREB signalling pathway. CREB showed decreased RNA expression in response to MALAT1 silence (Figure 7A). Additionally, consistent with the finding in preceding research, our Western blotting verified that CREB phosphorylation was depressed after siRNA‐MALAT1 transfection (Figure 7B). We performed a ChIP analysis for hyperoxia‐stimulated A549 cells. Silencing MALAT1 decreased the binding of CREB to STING promoter (Figure 7C). Therefore, MALAT1 can affect the mRNA expression, phosphorylation and binding (to STING promoter) of CREB.

**FIGURE 8:**
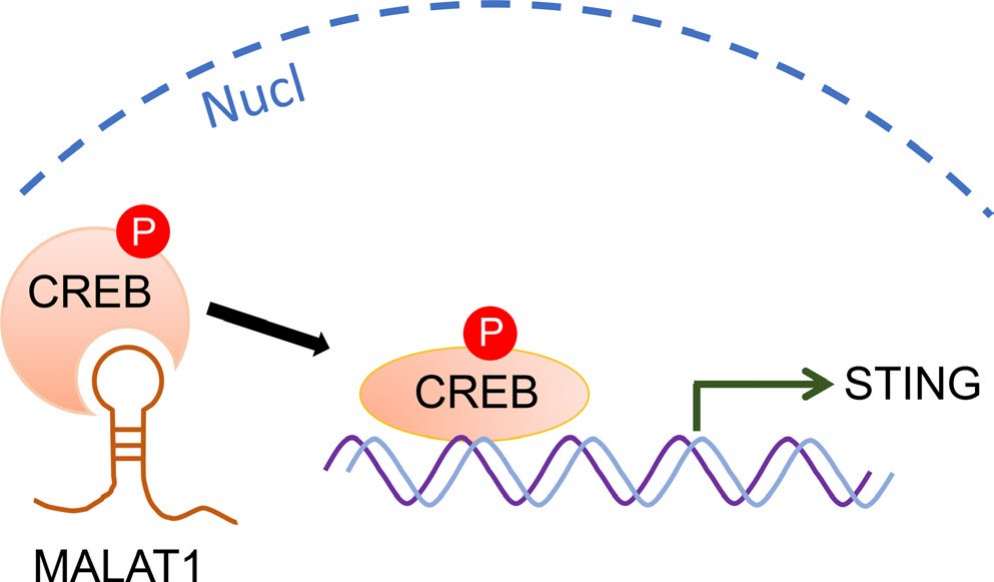
MALAT1 interacts with CREB to regulate STING transcription in BPD neonates. In the nucleus, MALAT1 contributes to the phosphorylation of CREB, then increases the binding (to the promoter of STING) of CREB and further triggers the transcription of STING

To confirm whether MALAT1‐induced STING promoter activity was dependent on CREB, we transfected the hyperoxia‐induced A549 and Beas‐2B cells with previously constructed plasmids containing mutated CREB‐binding sites. Then, the transfected cells were treated with siRNA‐MALAT1 for 48 hours before luciferase assay. We observed that the STING promoter activity was suppressed in wild‐type plasmids‐treated cells while there is no activity change in mutant CREB‐binding sites plasmids‐treated cells after MALAT1 silencing (Figure 7D). Herein, a mechanism map was constructed to elucidate the interaction among STING, MALAT1 and CREB (Figure 8). Based on these findings, we advocated that MALAT1 modulated the transcription of STING through MALAT1‐CREB signalling pathway.

## 4 DISCUSSION

Up to now, extensive research has shown that BPD has a multifactorial pathogenesis, especially imbalanced inflammation,^9^ which is considered to be a major underlying mechanism. A body of inflammation‐related factors and mediators is involved in the development of BPD, such as IL1β,^27^ NLRP3,^28^ angiotensinogen (AGT),^29^ IL‐6 ^30^ and tumour necrosis factor α (TNF‐α).^31^ Another factor is oxidative stress that may lead to the stimulation of inflammatory cells, activation of pro‐inflammatory cytokines, impairment or apoptosis of respiratory tract epithelium.^32^, ^33^

This is the first study to delve into the role of MALAT1 and STING in the pathogenesis of BPD. We collected PBMC specimens from 57 BPD newborns and unrelated healthy controls. We found that both STING and MALAT1 were significantly up‐regulated in BPD patients. Afterwards, we established a BPD model using neonatal rats exposed to hyperoxia. Compared with that of the normoxia group, the mRNA and protein levels of STING and MALAT1 expression in the hyperoxia group was gradually increased during Day 1‐21 after birth, which was also confirmed by the results of immunohistochemistry. We also detected the gene expression of STING and MALAT1 in hyperoxia‐induced lung cells. Results showed that the mRNA levels of both MALAT1 mRNA and STING were significantly increased after 48 hours of hyperoxia exposure and then decreased gradually, which was consistent with what we observed in BPD children and rat model. Given that, we hypothesized that MALAT1 may be relevant to STING in BPD development.

STING can activate interferons (IFN) regulatory factor 3 (IRF3) and nuclear factor (NF)‐κB transcription pathways to produce various cytokines, including type I IFNs and pro‐inflammatory cytokines.^34^ Recent evidence suggests that self‐DNA leaking from the nucleus of the host cells may also activate the STING pathway, giving rise to various inflammatory diseases including STING‐associated vasculopathy with onset in infancy (SAVI), Aicardi‐Goutières syndrome (AGS), systemic lupus erythematosus (SLE) and even inflammation‐associated cancer.^35^, ^36^ STING also acts as an essential sensor in chronic lung inflammation caused by long‐term exposure to airborne pollutants (eg cigarette smoke) or particulate toxicants (eg crystalline silica),^7^ asthma characterized by airway remodelling and hyper‐responsiveness,^37^ respiratory infection by viruses and bacteria and other lung diseases.^38^ Prior studies have noted the importance of STING in antiviral and antitumor function.^39^, ^40^ Additionally, it is now well established that STING is remarkably related with miscellaneous pulmonary diseases. Silencing STING may lead to primary resistance to immunotherapy in non–small‐cell lung cancer (NSCLC).^41^ STING is also engaged in the pathophysiological course of pulmonary tuberculosis through producing type I IFNs.^42^ Overactivated STING by gain‐of‐function mutations in *TMEM173* appears to relate to dysfunction of endothelium and causes interstitial lung disease in SAVI.^43^ However, no study used to investigate the impact of STING on BPD. It is elucidated that overexpression of STING triggers the apoptosis of primary and malignant T or B cells.^8^, ^44^, ^45^ In our study, the apoptosis of hyperoxia‐induced A549 and Beas‐2B cells were significantly decreased after STING knockdown and then increased after STING overexpression. CCK‐8 assay also confirmed that STING repressed the proliferation of hyperoxia‐induced A549 and Beas‐2B cells. Thus, it is indicated that STING may take part in the process of BPD via affecting the apoptosis and the proliferation of lung epithelial cells.

Dozens of lncRNAs can regulate gene expression in various biological processes, including apoptosis, proliferation, migration, differentiation, autophagy and pyroptosis.^46^, ^47^, ^48^ In a recent report, researchers have screened differentially expressed lncRNAs in a hyperoxia‐induced neonatal mouse BPD model, indicating that lncRNAs might participate in BPD development.^49^ MALAT1, one lncRNA, is involved in several physiopathological process including angiogenesis,^50^ diabetes progression,^51^ tumour progression,^52^ cardiovascular remodelling ^53^ and tissue inflammation.^19^ It can play multiple roles, like promoting protein localization, acting as a competing endogenous RNA, or regulating protein activity, gene transcription and epigenetic changes.^54^ Interestingly, it has been reported that MALAT1 expression was elevated in BPD patients, implying its linkage with BPD.^22^ Preceding research showed that MALAT1 could promote cell apoptosis and inhibit cell proliferation in various diseases.^20^, ^21^ Our results showed that knockdown of MALAT1 inhibited the apoptosis and promoted the proliferation of hyperoxia‐induced lung cells. Its performance showed accordance with that of STING, which further cemented the relevance between STING and MALAT1 in BPD. To explore the role of STING and MALAT1 in BPD, we suppressed MALAT1 at an optimum level in the cell models. As a consequence, the mRNA and protein levels of STING were down‐regulated by after transfection with siRNA‐MALAT1 in A549 and Beas‐2B cells, compared with those transfected with siRNA‐NC. What's more, the promoter activity of STING was detected to be decreased when cotransfected siRNA‐MALAT1 and pGL‐126/+1 plasmid, suggestive of the positive correlation between MALAT1 and STING.

CREB, a classical transcription factor, was found mainly involving in the synchronization of circadian rhythmicity and the synthesis of many neurotrophic factors. Its implication in Alzheimer's disease (AD) has been verified.^12^ Besides, it can also modulate basic metabolic processes, including glucose metabolism, fatty acid oxidation and hepatic lipid mobilization.^55^ CREB‐binding protein (CBP) and p300 are recruited upon the phosphorylation of CREB at Ser‐133 (p‐CREB), allowing the transcription of target genes.^12^ Our previous research has found out the cooperative mechanism between CREB and STING, and then, we advanced to prove this mechanism in BPD. As expected, the present study identified that overexpression of CREB increased the promoter activity, mRNA and protein expression of STING gene, which were all reversed after CREB expression was repressed. Next, our ChIP results showed that CREB could directly bind to the promoter of STING to regulate its transcription. Based on these data, we announce that CREB can regulate the promoter activity and expression of STING and that hyperoxia‐induced STING transcription may depend on CREB.

LncRNA MALAT1 can affect gene transcription through forming RNA‐protein complexes with proteins.^54^ For example, investigators found that MALAT1 inhibited NF‐κB activity via preventing p65 it from binding to target DNA.^56^ Another study also showed that MALAT1 inhibited CD80 transcription by interfering with the binding of transcription factor NF‐κB to CD80 promoter.^57^ Hence, we hypothesized that down‐regulating MALAT1 may modulate CREB expression and phosphorylation, and its binding to STING. According to the results, CREB expression was decreased after MALAT1 knockdown and CREB phosphorylation was inhibited after siRNA‐MALAT1 transfection. Moreover, ChIP assay also showed that silencing MALAT1 curbed the binding of CREB to STING promoter. Meanwhile, we found that mutation of CREB binding sites relieved the suppression on STING promoter activity after 48 hours of MALAT1 siRNA transfection. Based on these findings, we propose that MALAT1 can regulate the transcription of STING through MALAT1‐CREB signalling pathway.

In conclusion, MALAT1 interacts with CREB to regulate STING transcription in BPD neonates. STING, CREB and MALAT1 may be promising therapeutic targets in the prevention and treatment of BPD.

## CONFLICT OF INTEREST

The authors confirm that there is no conflict of interests.

## AUTHOR CONTRIBUTION

**Jiahe he Chen:** Conceptualization (lead); Data curation (lead); Formal analysis (lead); Investigation (lead); Methodology (lead); Resources (lead); Software (equal); Supervision (equal); Validation (lead); Visualization (lead); Writing‐original draft (lead); Writing‐review & editing (lead). **Dandan Feng:** Conceptualization (supporting); Data curation (supporting); Formal analysis (supporting); Investigation (supporting); Methodology (supporting); Project administration (supporting); Software (supporting); Supervision (supporting); Validation (supporting); Visualization (supporting); Writing‐review & editing (supporting). **Yufei Chen:** Data curation (supporting); Formal analysis (supporting); Resources (supporting); Software (supporting); Writing‐review & editing (supporting). **Caixia Yang:** Conceptualization (supporting); Data curation (supporting); Formal analysis (supporting); Methodology (supporting). **Chenxia Juan:** Investigation (supporting); Project administration (supporting). **Qian Cao:** Conceptualization (supporting); Data curation (supporting); Investigation (supporting); Methodology (supporting); Writing‐review & editing (supporting). **Xi Chen:** Data curation (supporting); Investigation (supporting); Methodology (supporting). **Shuang Liu:** Data curation (supporting); Formal analysis (supporting); Methodology (supporting). **Guoping Zhou:** Conceptualization (supporting); Data curation (supporting); Formal analysis (supporting); Funding acquisition (lead); Investigation (supporting); Methodology (supporting); Project administration (lead); Resources (supporting); Software (supporting); Supervision (supporting); Validation (supporting); Visualization (supporting); Writing‐original draft (supporting); Writing‐review & editing (supporting).

## Figures and Tables

**FIGURE A1:**
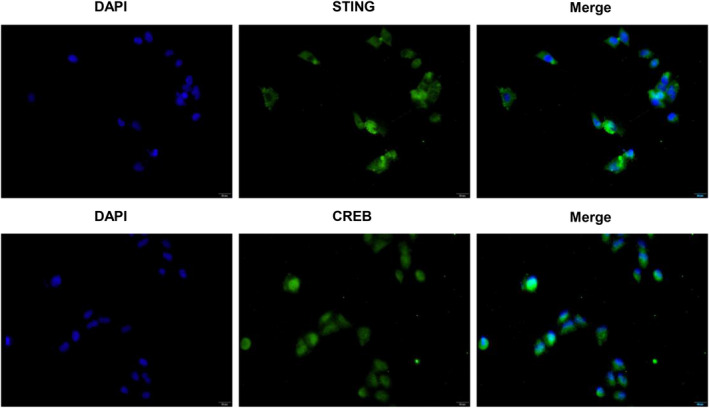
The location of STING and CREB in cells. Through the cell immunofluorescence, STING was located mainly in the cytoplasm while CREB was located in both cytoplasm and nucleus.

## Data Availability

The data used to support the findings of this study are available from the corresponding author upon request.

## References

[1] Kirpalani H , Ratcliffe SJ , Keszler M , et al. Effect of sustained inflations vs intermittent positive pressure ventilation on bronchopulmonary dysplasia or death among extremely preterm infants: the SAIL Randomized Clinical Trial. JAMA. 2019;321:1165‐1175. 30912836 10.1001/jama.2019.1660 PMC6439695

[2] Hastrup LH , Jennum P , Ibsen R , et al. Societal costs of Borderline Personality Disorders: a matched‐controlled nationwide study of patients and spouses. Acta Psychiatr Scand. 2019;140:458‐467. 31483859 10.1111/acps.13094

[3] Tatler AL , Barnes J , Habgood A , et al. Caffeine inhibits TGFbeta activation in epithelial cells, interrupts fibroblast responses to TGFbeta, and reduces established fibrosis in ex vivo precision‐cut lung slices. Thorax. 2016;71:565‐567. 26911575 10.1136/thoraxjnl-2015-208215 PMC4893128

[4] Thébaud B , Ladha F , Michelakis ED , et al. Vascular endothelial growth factor gene therapy increases survival, promotes lung angiogenesis, and prevents alveolar damage in hyperoxia‐induced lung injury: evidence that angiogenesis participates in alveolarization. Circulation. 2005;112:2477‐2486. 16230500 10.1161/CIRCULATIONAHA.105.541524

[5] Ablasser A . Structures of STING protein illuminate this key regulator of inflammation. Nature. 2019;567:321‐322. 30880329 10.1038/d41586-019-00707-8

[6] Barber GN . STING: infection, inflammation and cancer. Nat Rev Immunol. 2015;15:760‐770. 26603901 10.1038/nri3921 PMC5004891

[7] Benmerzoug S , Ryffel B , Togbe D , et al. Self‐DNA sensing in lung inflammatory diseases. Trends Immunol. 2019;40:719‐734. 31262653 10.1016/j.it.2019.06.001

[8] Gulen MF , Koch U , Haag SM , et al. Signalling strength determines proapoptotic functions of STING. Nat Commun. 2017;8:427. 28874664 10.1038/s41467-017-00573-w PMC5585373

[9] Dong Y , Speer CP , Glaser K . Beyond sepsis: Staphylococcus epidermidis is an underestimated but significant contributor to neonatal morbidity. Virulence. 2018;9:621‐633. 29405832 10.1080/21505594.2017.1419117 PMC5955464

[10] Das KC , Wasnick JD . Biphasic response of checkpoint control proteins in hyperoxia: exposure to lower levels of oxygen induces genome maintenance genes in experimental baboon BPD. Mol Cell Biochem. 2014;395:187‐198. 24939362 10.1007/s11010-014-2124-1 PMC4172380

[11] Mayr B , Montminy M . Transcriptional regulation by the phosphorylation‐dependent factor CREB. Nat Rev Mol Cell Biol. 2001;2:599‐609. 11483993 10.1038/35085068

[12] Bartolotti N , Bennett DA , Lazarov O . Reduced pCREB in Alzheimer's disease prefrontal cortex is reflected in peripheral blood mononuclear cells. Mol Psychiatry. 2016;21:1158‐1166. 27480489 10.1038/mp.2016.111 PMC4995548

[13] Hu Z , Han Y , Liu Y , et al. CREBZF as a key Regulator of STAT3 Pathway in the Control of Liver Regeneration in Mice. Hepatology. 2020;71(4):1421‐1436. 31469186 10.1002/hep.30919

[14] Alexaki VI , Fodelianaki G , Neuwirth A , et al. DHEA inhibits acute microglia‐mediated inflammation through activation of the TrkA‐Akt1/2‐CREB‐Jmjd3 pathway. Mol Psychiatry. 2018;23:1410‐1420. 28894299 10.1038/mp.2017.167

[15] Thornton CC , Al‐Rashed F , Calay D , et al. Methotrexate‐mediated activation of an AMPK‐CREB‐dependent pathway: a novel mechanism for vascular protection in chronic systemic inflammation. Ann Rheum Dis. 2016;75:439‐448. 25575725 10.1136/annrheumdis-2014-206305 PMC4752671

[16] Yao J , Wang XQ , Li YJ , et al. Long non‐coding RNA MALAT1 regulates retinal neurodegeneration through CREB signaling. EMBO Mol Med. 2016;8:1113. 27587362 10.15252/emmm.201606749 PMC5009814

[17] Engreitz JM , Ollikainen N , Guttman M . Long non‐coding RNAs: spatial amplifiers that control nuclear structure and gene expression. Nat Rev Mol Cell Biol. 2016;17:756‐770. 27780979 10.1038/nrm.2016.126

[18] Zhang X , Hamblin MH , Yin KJ . The long noncoding RNA Malat 1: Its physiological and pathophysiological functions. RNA Biol. 2017;14:1705‐1714. 28837398 10.1080/15476286.2017.1358347 PMC5731810

[19] Cremer S , Michalik KM , Fischer A , et al. Hematopoietic deficiency of the long noncoding RNA MALAT1 promotes atherosclerosis and plaque inflammation. Circulation. 2019;139:1320‐1334. 30586743 10.1161/CIRCULATIONAHA.117.029015

[20] Li W , Ning J‐Z , Cheng F , et al. MALAT1 promotes cell apoptosis and suppresses cell proliferation in testicular ischemia‐reperfusion injury by sponging MiR‐214 to modulate TRPV4 expression. Cell Physiol Biochem. 2018;46:802‐814. 29870987 10.1159/000488738

[21] Wu Q , Yi X . Down‐regulation of long noncoding RNA MALAT1 protects hippocampal neurons against excessive autophagy and apoptosis via the PI3K/Akt signaling pathway in rats with epilepsy. J Mol Neurosci. 2018;65:234‐245. 29858824 10.1007/s12031-018-1093-3

[22] Cai C , Qiu J , Qiu G , et al. Long non‐coding RNA MALAT1 protects preterm infants with bronchopulmonary dysplasia by inhibiting cell apoptosis. BMC Pulm Med. 2017;17:199. 29237426 10.1186/s12890-017-0524-1 PMC5729463

[23] Jobe AH , Bancalari E . Bronchopulmonary dysplasia. Am J Respir Crit Care Med. 2001;163:1723‐1729. 11401896 10.1164/ajrccm.163.7.2011060

[24] Thébaud B , Goss KN , Laughon M , et al. Bronchopulmonary dysplasia. Nat Rev Dis Primers. 2019;5:78. 31727986 10.1038/s41572-019-0127-7 PMC6986462

[25] Cooney TP , Thurlbeck WM . The radial alveolar count method of Emery and Mithal: a reappraisal 1–postnatal lung growth. Thorax. 1982;37:572‐579. 7179185 10.1136/thx.37.8.572 PMC459377

[26] Syed M , Das P , Pawar A , et al. Hyperoxia causes miR‐34a‐mediated injury via angiopoietin‐1 in neonatal lungs. Nat Commun. 2017;8:1173. 29079808 10.1038/s41467-017-01349-y PMC5660088

[27] Zhao W , Ma L , Cai C , et al. Caffeine inhibits NLRP3 inflammasome activation by suppressing MAPK/NF‐kappaB and A2aR signaling in LPS‐induced THP‐1 macrophages. Int J Biol Sci. 2019;15:1571‐1581. 31360100 10.7150/ijbs.34211 PMC6643212

[28] Liao J , Kapadia VS , Brown LS , et al. The NLRP3 inflammasome is critically involved in the development of bronchopulmonary dysplasia. Nat Commun. 2015;6:8977. 26611836 10.1038/ncomms9977 PMC6215764

[29] Shen L , Zhang T , Lu H . Overexpression of AGT promotes bronchopulmonary dysplasis via the JAK/STAT signal pathway. Oncotarget. 2017;8:96079‐96088. 29221188 10.18632/oncotarget.21712 PMC5707082

[30] Hsiao C‐C , Chang J‐C , Tsao L‐Y , et al. Correlates of Elevated Interleukin‐6 and 8‐Hydroxy‐2'‐Deoxyguanosine Levels in Tracheal Aspirates from Very Low Birth Weight Infants Who Develop Bronchopulmonary Dysplasia. Pediatr Neonatol. 2017;58:63‐69. 27321203 10.1016/j.pedneo.2016.01.004

[31] Özdemir ÖMA , Taban Ö , Enli Y , et al. The effects of bosentan on hyperoxia‐induced lung injury in neonatal rats. Pediatr Int. 2019;61:1120‐1126. 31560816 10.1111/ped.14013

[32] Zhang X , Chu X , Gong X , et al. The expression of miR‐125b in Nrf2‐silenced A549 cells exposed to hyperoxia and its relationship with apoptosis. J Cell Mol Med. 2020;24:965‐972. 31713992 10.1111/jcmm.14808 PMC6933325

[33] Amata E , Pittalà V , Marrazzo A , et al. Role of the Nrf2/HO‐1 axis in bronchopulmonary dysplasia and hyperoxic lung injuries. Clin Sci (Lond). 2017;131:1701‐1712. 28667068 10.1042/CS20170157

[34] Cai X , Chiu YH , Chen ZJ . The cGAS‐cGAMP‐STING pathway of cytosolic DNA sensing and signaling. Mol Cell. 2014;54:289‐296. 24766893 10.1016/j.molcel.2014.03.040

[35] Ahn J , Barber GN . Self‐DNA, STING‐dependent signaling and the origins of autoinflammatory disease. Curr Opin Immunol. 2014;31:121‐126. 25459004 10.1016/j.coi.2014.10.009

[36] Li N , Zhou H , Wu H , et al. STING‐IRF3 contributes to lipopolysaccharide‐induced cardiac dysfunction, inflammation, apoptosis and pyroptosis by activating NLRP3. Redox Biol. 2019;24:101215. 31121492 10.1016/j.redox.2019.101215 PMC6529775

[37] Carroll EC , Jin L , Mori A , et al. The vaccine adjuvant chitosan promotes cellular immunity via DNA sensor cGAS‐STING‐dependent induction of type I interferons. Immunity. 2016;44:597‐608. 26944200 10.1016/j.immuni.2016.02.004 PMC4852885

[38] Marinho FV , Benmerzoug S , Oliveira SC , et al. The emerging roles of STING in bacterial infections. Trends Microbiol. 2017;25:906‐918. 28625530 10.1016/j.tim.2017.05.008 PMC5650497

[39] Hoyland‐Kroghsbo NM . Cyclic nucleotide signaling: a second messenger of death. Cell Host Microbe. 2019;26:567‐568. 31726022 10.1016/j.chom.2019.10.017

[40] You H , Lin Y , Lin F , et al. Beta‐catenin is required for cGAS/STING signaling pathway but antagonized by HSV‐1 US3 protein. J Virol. 2020;94(5):e01847‐19. 31801859 10.1128/JVI.01847-19 PMC7022340

[41] Della Corte CM , Byers LA . Evading the STING: LKB1 loss leads to STING silencing and immune escape in KRAS‐mutant lung cancers. Cancer Discov. 2019;9:16‐18. 30626603 10.1158/2159-8290.CD-18-1286 PMC8330553

[42] Bénard A , Sakwa I , Schierloh P , et al. B cells producing type I IFN modulate macrophage polarization in tuberculosis. Am J Respir Crit Care Med. 2018;197:801‐813. 29161093 10.1164/rccm.201707-1475OC PMC5855072

[43] Liu Y , Jesus AA , Marrero B , et al. Activated STING in a vascular and pulmonary syndrome. N Engl J Med. 2014;371:507‐518. 25029335 10.1056/NEJMoa1312625 PMC4174543

[44] Tang C‐H , Zundell JA , Ranatunga S , et al. Agonist‐mediated activation of STING induces apoptosis in malignant B cells. Cancer Res. 2016;76:2137‐2152. 26951929 10.1158/0008-5472.CAN-15-1885 PMC4873432

[45] Zierhut C , Yamaguchi N , Paredes M , et al. The cytoplasmic DNA sensor cGAS promotes mitotic cell death. Cell. 2019;178(2):302‐315.e23. 31299200 10.1016/j.cell.2019.05.035 PMC6693521

[46] Li X , Zeng LI , Cao C , et al. Long noncoding RNA MALAT1 regulates renal tubular epithelial pyroptosis by modulated miR‐23c targeting of ELAVL1 in diabetic nephropathy. Exp Cell Res. 2017;350:327‐335. 27964927 10.1016/j.yexcr.2016.12.006

[47] Mendell JT . Targeting a long noncoding RNA in breast cancer. N Engl J Med. 2016;374:2287‐2289. 27276568 10.1056/NEJMcibr1603785

[48] Wang J , Yin J , Wang X , et al. Changing expression profiles of mRNA, lncRNA, circRNA, and miRNA in lung tissue reveal the pathophysiological of bronchopulmonary dysplasia (BPD) in mouse model. J Cell Biochem. 2019;120:9369‐9380. 30802330 10.1002/jcb.28212

[49] Bao T‐P , Wu R , Cheng H‐P , et al. Differential expression of long non‐coding RNAs in hyperoxia‐induced bronchopulmonary dysplasia. Cell Biochem Funct. 2016;34:299‐309. 27137150 10.1002/cbf.3190

[50] Michalik KM , You X , Manavski Y , et al. Long noncoding RNA MALAT1 regulates endothelial cell function and vessel growth. Circ Res. 2014;114:1389‐1397. 24602777 10.1161/CIRCRESAHA.114.303265

[51] Liu J‐Y , Yao J , Li X‐M , et al. Pathogenic role of lncRNA‐MALAT1 in endothelial cell dysfunction in diabetes mellitus. Cell Death Dis. 2014;5:e1506. 25356875 10.1038/cddis.2014.466 PMC4649539

[52] Kim J , Piao H‐L , Kim B‐J , et al. Long noncoding RNA MALAT1 suppresses breast cancer metastasis. Nat Genet. 2018;50:1705‐1715. 30349115 10.1038/s41588-018-0252-3 PMC6265076

[53] Puthanveetil P , Gutschner T , Lorenzen J . MALAT1: a therapeutic candidate for a broad spectrum of vascular and cardiorenal complications. Hypertens Res. 2020;43(5):372‐379. 31853043 10.1038/s41440-019-0378-4

[54] Lei LI , Chen J , Huang J , et al. Functions and regulatory mechanisms of metastasis‐associated lung adenocarcinoma transcript 1. J Cell Physiol. 2018;234:134‐151. 30132842 10.1002/jcp.26759

[55] Roy A , Jana M , Kundu M , et al. HMG‐CoA reductase inhibitors bind to PPARalpha to upregulate neurotrophin expression in the brain and improve memory in mice. Cell Metab. 2015;22:253‐265. 26118928 10.1016/j.cmet.2015.05.022 PMC4526399

[56] Zhao G , Su Z , Song D , et al. The long noncoding RNA MALAT1 regulates the lipopolysaccharide‐induced inflammatory response through its interaction with NF‐kappaB. FEBS Lett. 2016;590:2884‐2895. 27434861 10.1002/1873-3468.12315

[57] Juan C , Wang Q , Mao Y , et al. Knockdown of LncRNA MALAT1 contributes to cell apoptosis via regulating NF‐kappaB/CD80 axis in neonatal respiratory distress syndrome. Int J Biochem Cell Biol. 2018;104:138‐148. 30243953 10.1016/j.biocel.2018.09.009

